# Synthesis and Antiulcer Activity Studies of 2-(1′-Iminothioimido Substituted)-1′-Substituted Phenylbenzoic acids

**DOI:** 10.4103/0250-474X.43009

**Published:** 2008

**Authors:** B. B. Subudhi, D. Bhatta, P. K. Panda, P. Mishra, D. Pradhan

**Affiliations:** University Department of Pharmaceutical Sciences, Utkal University, Vanivihar, Bhubaneshwar-751 004, India

**Keywords:** Benzothiazole, benzotriazole, benzimidazole, total acidity, free acidity, ulcer index

## Abstract

Certain 2-(1'-iminothioimido substituted)-1'-substituted phenybenzoic acids (P_1-9_) were synthesized by reaction of phthalic anhydride with benzotriazole, 2-mercapto benzothiazole and 2-p-amino phenyl benzimidazole, respectively (A_1-3_) followed by imine formation with Schiff bases of thiourea with salicylaldehyde, furfuraldehyde and 1-phenyl-3-methyl-5-pyrazolone. Antiulcer activity was evaluated using reduction in total acidity, free acidity and ulcer index as parameters. Compounds P_3_, P_6_, P_7_ and P_9_(100 mg/kg) showed significant (P< 0.001) antiulcer action compared to control and omeprazole (40 mg/kg).

The long-term acid suppression by irreversible H^+^/K^+^-ATPase inhibitors because of covalent binding to the specific enzyme is reported to increase *C. difficalies* infection^1^, improper absorption of Vit-B_12_^2^, calcium^3^ and other opportunistic infections^4^. SCH 28080^5^ is the prototype of a reversible proton pump inhibitor, which is effective in ulcer healing yet avoid prolonged achlorhydria. Derivatives of imidazole^6^ quinoline^7^, isoquinoline^8^, quinazoline^9^, pyrazine^10^ and thiazole^11^ that lack the sulfinyl group but contain a basic group have been reported as reversible proton pump inhibitors. Keeping in view this structural flexibility and capitalizing on the basic structural feature, we thought to explore several benzimidazole, benzotriazole and benzothiazole derivatives to find some alternatives to the present irreversible proton pump inhibitors.

With the above aim, different 2-(1'-iminothioimido substituted)-1'-substituted phenyl benzoic acids (P_1-9_) were prepared. Melting points were determined in open capillary tubes and are uncorrected. The purity of the compounds was checked on silica gel-G coated plates using iodine as visualizing agent. Infrared spectra were recorded on Perkin Elmer model 600 spectrophotometer using KBr pellet. ^1^H NMR (DMSO d-_6_) spectra of title compounds were recorded on a Bruker DRX-300 NMR spectrophotometer (300 MHz) using TMS as internal standard. The C, H and N elemental analysis was carried out using a Perkin Elmer-2400 elemental analyser. The animal experiments were carried out following the protocols approved by the Institutional Animal Ethics Committee of the University Department of Pharmaceutical Sciences, Utkal University (Registration no-990/c/06/CPCSEA). The title compounds (P _1-9_) at a dose of 100 mg/kg were tested for antiulcer activity^12^ using reduction in total acidity, free acidity and ulcer index as parameters. Omeprazole (40 mg/kg) was used as the standard drug.

The compounds (A _1-3_) were prepared according to standard procedure (Fig. 1)^13^. Schiff bases of thiourea with salicylaldehyde, furfuraldehyde and 1-phenyl-3-methyl-5-pyrazolone were synthesized as per the standard laboratory method^14^. A mixture of compound A (0.015 mol) and corresponding Schiff base (0.015 mol) were taken in 25 ml of ethanol. Few drops of glacial acetic acid were added to it. The mixture was refluxed for 2 h. It was allowed to cool and then mixed with ice-cold water. The precipitate was filtered, dried, and recrystallised with 1, 4-dioxan. Nine such compounds P_(1-9)_ were synthesized and characterized (Table 1). Following the above procedure compound 2-(1'-imino thioimido-o-hydroxy benzyl)-1'-(2”-mercaptobenzthiazolyl) benzoic acid (P_5_) was obtained as a crystalline product. IR (KBr, cm^-1^) spectrum of compound P_5_ exhibited bands at 3366 cm^-1^, (O-H), 1469 cm^-1^ (C=C, ring of benzene), 1632 cm^-1^ (C=O) and 1511 cm^-1^ (C=N).^1^H NMR (DMSO d-_6_, δ ppm) spectra of P_5_ exhibited peaks at δ 7.0-7.5(m, 4H, Ar-H), δ 11.5(s, 1H, -OH), δ 8.1(s, 1H, CH=N). Other compounds of the series P_1-9_ displayed broad IR absorption bands corresponding to Ar-OH superimposed on that of NH in the range of 3285-3368 cm^-1^. Another sharp band appeared at 1645-1687 cm^-1^ due to carbonyl function. Bands assigned to stretching vibrations of carbon-carbon double bonds of aromatic ring were observed at 1442-1489 cm^-1^. ^1^H NMR (DMSO d-_6_, δ ppm) spectral studies exhibited a broad singlet at δ 11.7-13.2 due to proton of aromatic carboxylic acid group. Another broad singlet appeared at δ 4.8-5.45 due to N-H proton. The aromatic protons appeared as multiplets in the range δ 7.05-7.65.

**Fig. 1:**
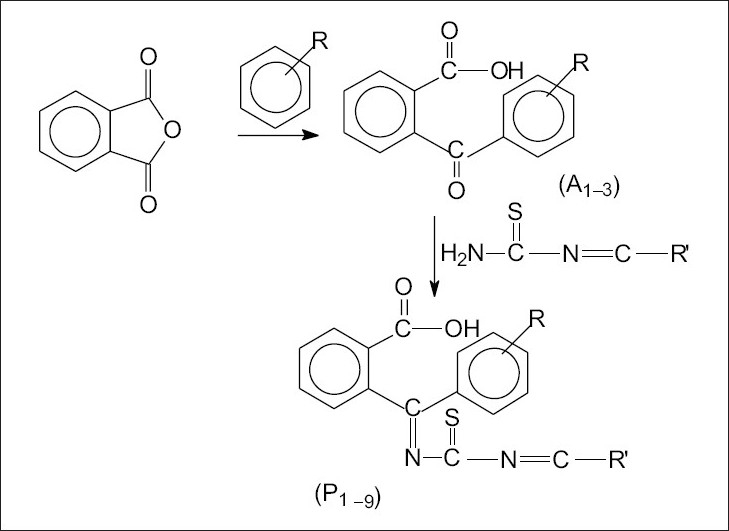
Scheme of the synthesized compound. For compound P_1_, R is p-amino phenyl-2-imidazole and R′ is 1-phenyl-3-methyl-pyrazole; for compound P_2_, R is p-amino phenyl- 2-imidazole and R′ is 2-hydroxy phenyl; for compound P_3_, R is p-amino phenyl-2-imidazole and R′ is furfuryl; for compound P_4_, R is 2-mercapto thiazole and R′ is 1-phenyl-3-methyl-pyrazole; for compound P_5_, R is 2-mercapto thiazole and R′ is 2-hydroxy phenyl; for compound P_6_, R is 2-mercapto thiazole and R′ is furfuryl; for compound P_7_ R is triazole and R′ is1-phenyl-3-methyl-pyrazole; for compound P_8_, R is triazole and R′ is 2-hydroxy phenyl and for compound P_9_, R is triazole and R′ is furfuryl.

**TABLE 1:** PHYSICAL AND ANALYTICAL DATA OF COMPOUNDS P_(1-9)_

Compd	Mol. Formula	Mol. Wt.	Yield %	mp	% of C, H, N calculated (found)		
					
					C	H	N
P_1_	C_32_H_25_N_7_O_2_S	571.1	51.59	167	67.25(68.12)	4.37(4.28)	17.15(17.89)
P_2_	C_29_H_21_N_5_O_3_S	519.1	54.23	162	67.03(67.45)	4.04(4.26)	13.48(13.22)
P_3_	C_27_H_19_N_5_O_3_S	493.1	48.82	179	65.70(66.1)	3.85(4.21)	14.19(14.32)
P_4_	C_26_H_19_N_5_O_2_S_3_	529.3	47.72	155	58.94(59.12)	3.58 (4.23)	13.22(13.20)
P_5_	C_23_H_15_N_3_O_3_S_3_	477.3	53.16	176	57.82(57.80)	3.14(3.28)	8.79(9.21)
P_6_	C_21_H_13_N_3_O_3_S_3_	451.3	46.98	173	55.83(55.13)	2.88(2.67)	9.30 (9.54)
P_7_	C_25_H_19_N_7_O_2_S	481.1	51.49	159	62.35(63.08)	3.94(4.01)	20.36(20.81)
P_8_	C_22_H_15_N_5_O_3_S	429.1	55.48	170	61.52(61.29)	3.49(3.45)	16.31(16.95)
P_9_	C_20_H_13_N_5_O_3_S	403.1	58.74	184	59.53(59.24)	3.22(3.14)	17.36(17.69)

Male rats weighing between 140 and 175 g were selected for pyloric ligation ulcer model^12^. Rats were divided into eleven groups consisting of six animals each and were fasted overnight. One group received normal saline 2 ml/kg (negative control). The second group received omeprazole 40 mg/kg (positive control) and the other groups received test compounds (100 mg/kg) by oral route 30 min prior to pyloric ligation. Animals were sacrificed 4 h later and the stomach was opened to collect the gastric contents. The gastric contents were centrifuged at 1000 rpm for 10 min. One ml of the supernatant liquid was pipetted out and diluted to 10 ml with distilled water. The solution was titrated against 0.01 N sodium hydroxide solution using Topfer's reagent as indicator to the end point when the solution turned to orange color. The volume of sodium hydroxide consumed was taken as corresponding to the free acidity. Titration was further continued till the solution regained pink color. The volume of sodium hydroxide solution required was noted and total acidity calculated. After opening the stomach the ulcer index was calculated. The results are expressed as mean±SEM. The difference between groups was determined using the one way analysis of variance (ANOVA) followed by Dunnett's test and p< 0.05 was considered significant.

From the data in Table 2 it can be inferred that all the compounds at the tested dose level exhibited varying degree of antiulcer activity. The compound P_3_, P_6_, P_7_ and P_9_ exhibited good gastro protective actions as indicated by their very low ulcer index, free acidity and total acidity values. The inhibition of gastric acid secretion was better for benzotriazole and benzothiazole derivatives than substituted benzimidazoles. However benzimidazole derivatives exhibited significant capacity for neutralization of gastric acid compared to control. The present study indicates the necessity of a basic group but shows structural flexibility among the compounds to exert the antiulcer effect. Presence of iminothioimido derivatives of substituted pyrazole, fufuryl and o-hydroxy phenyl does not seem to affect activity significantly.

**TABLE 2:** ANTIULCER ACTIVITY DATA OF COMPOUNDS P_(1-9)_

Compound	Total acid (mEq/l)	Free acid (mEq/l)	Ulcer index
Control	11.4± 1.6	3.18±0.29	4.4±0.35
Omeprazole	3.2± 1.25*	0.42±0.25*	0.33±0.06*
P_1_	8.69±0.21*	2.52±0.42*	3.66±0.81*
P_2_	8.31±0.29*	2.18±0.38*	3.36±0.65*
P_3_	6.4±0.75*	2.11±0.30*	2.33±0.22*
P_4_	9.04±0.34*	2.92±0.12*	3.4±0.29*
P_5_	7.3±0.57*	2.41±0.17*	2.83±0.34*
P_6_	4.8±0.55*	1.79±0.08*	1.41±0.14*
P_7_	5.02±0.35*	1.77±0.23*	2.33±0.30*
P_8_	8.21±0.34*	2.62±0.27*	3.17±0.27*
P_9_	6.04±0.15*	2.13±0.02*	1.75±0.51*

Each value represents the mean± SEM. No of animals in each group were 6.

* p<0.05 as compared to control (Dunnett's test)
